# *GhNRPB3* Negatively Regulates Drought and Salt Tolerance in Cotton

**DOI:** 10.3390/plants14162575

**Published:** 2025-08-19

**Authors:** Yi Wang, Jiacong Zeng, Yuehua Yu, Zhiyong Ni

**Affiliations:** 1 Xinjiang Key Laboratory for Ecological Adaptation and Evolution of Extreme Environment Organisms, College of Life Sciences, Xinjiang Agricultural University, Urumqi 830052, China; wangyi604987664@126.com (Y.W.); zjc2855@163.com (J.Z.); 2 College of Agronomy, Xinjiang Agricultural University, Urumqi 830052, China

**Keywords:** RNA polymerase II, RPB3, cotton, drought, salt

## Abstract

RNA polymerase II (Pol II) has been shown to participate in various biological processes in plants, but its function in response to abiotic stress in cotton remains unclear. This study aimed to elucidate the role of the third-largest subunit of Pol II (NRPB3) in the response of cotton to drought and salt stress through molecular biology and physiological methods. Real-time fluorescence quantitative PCR was used to analyze the expression pattern of *GhNRPB3* in roots, stems, leaves, and cotyledons and to detect changes in its expression under drought, NaCl, and ABA treatments. Using virus-induced gene silencing (VIGS) technology, *GhNRPB3*-silenced plants were obtained, and their physiological indicators under drought and salt stress, as well as the expression levels of the drought stress-related genes *GhRD22* and *GhRD26*, were measured. This study revealed that *GhNRPB3* is widely expressed in roots, stems, leaves, and cotyledons and that its expression is significantly induced by drought, NaCl, and ABA treatments. Compared to wild-type plants, the drought resistance, survival rate, and peroxidase activity of the *GhNRPB3*-silenced plants significantly increased, whereas the malondialdehyde content significantly decreased. Moreover, the expression levels of the drought-responsive genes *GhRD22* and *GhRD26* significantly increased. The salt tolerance of the *GhNRPB3*-silenced plants also increased, as reflected by decreased leaf wilting and significant increases in root growth parameters (including root length, root area, and root volume). These results indicate that *GhNRPB3* plays a crucial role in mediating the adaptation of cotton to drought and salt stress by regulating the expression of stress-related genes.

## 1. Introduction

In eukaryotic cell nuclei, gene transcription is performed by three related multisubunit RNA polymerases, namely, RNA polymerase I, II, and III (Pol I, Pol II, and Pol III, respectively) [1]. Pol I consists of 14 subunits and is responsible for transcribing precursor rRNA, whereas Pol III consists of 17 subunits and is responsible for transcribing tRNAs and other small RNAs. Pol II is composed of 12 subunits (RPB1–RPB12), and it is the core enzyme that is responsible for transcribing protein-coding genes (generating mRNA precursors) and some noncoding RNAs (such as small nuclear RNAs (snRNAs) and microRNAs). The precise regulation of the structure and function of Pol II is crucial for gene expression networks [1].

Similar to Pol II in other eukaryotes, plant Pol II is a multisubunit complex composed of 12 subunits. The process of transcription begins with the formation of the core scaffold RPB3–RPB11 heterodimer, which then collaborates with structural modules that are composed of RPB2, RPB10, and RPB12 to form the basic structural framework [2]. Under the regulation of molecular chaperone systems, such as Hsp90/R2TP complexes, the largest catalytic subunit, RPB1, completes correct folding and precise integration, resulting in the formation of a core structure that has catalytic activity [3]. The final stage involves the gradual recruitment of regulatory subunits (including the RPB4–RPB7 dimer and auxiliary components such as RPB5, RPB6, RPB8, and RPB9) to complete the assembly of the fully functional enzyme complex [4,5]. This multistep collaborative assembly mechanism ensures the accuracy and functional integrity of the Pol II structure. Among these subunits, the third subunit of Pol II (RPB3) plays a central role in the entire assembly process [6].

Recent studies have shown that RPB3 not only plays a core role in Pol II holoenzyme assembly but also directly participates in the regulation of gene transcription processes. RPB3 regulates muscle cell differentiation and liver tumorigenesis by directly interacting with ATF4, myogenin, and Snail [7,8,9]. Moreover, RPB3 can specifically regulate the 3′ end processing of ribosomal protein-encoding genes via CBC–PCF11 and small elongation complexes [10]. Additionally, the oncogene EWS regulates the transcriptional activity of Pol II by directly binding to RPB3 and RPB5 [11]. In plants, a homolog of RPB3, namely, NRPB3, can interact with FAMA, ICE1, and RIMA to regulate stomatal development [12,13]. In addition, RPB3 plays important roles in plant growth and development. For example, mutations in RPB3 in *Arabidopsis* lead to plant death [12]. Although RPB3 has been confirmed to be involved in various transcriptional regulatory processes, there have been no reports on the function of this gene in the plant abiotic stress response.

Cotton, which is an important economic crop, is facing severe challenges in terms of its growth and development because of drought and salt stress caused by global climate change. Elucidating the molecular mechanism underlying drought and salt tolerance in cotton is crucial for overcoming the current technological bottleneck in the breeding of stress-resistant plants. Previous studies have shown that the expression of *GhMYB4* significantly increases the tolerance of cotton to drought and salt stress [14]. Through yeast library screening and yeast two-hybrid experiments, we identified GhNRPB3, namely, the third subunit of Pol II, and demonstrated its ability to form protein interactions with GhMYB4 [15]. These findings suggest that *GhNRPB3* may also play a key role in drought and salt tolerance in cotton.

This study revealed that the *GhNRPB3* gene is highly expressed in cotton cotyledon tissues and that its expression patterns differ under different abiotic stress conditions. After the expression of *GhNRPB3* was downregulated through VIGS, the *GhNRPB3*-knockdown cotton plants exhibited increased tolerance to drought and salt stress. Additionally, under drought stress conditions, the expression levels of the stress-responsive genes *GhRD22* and *GhRD26* were significantly upregulated in the silenced plants. These findings suggest that *GhNRPB3* may function as a negative regulatory factor in the response of cotton to drought and salt stress.

## 2. Results

### 2.1. GhNRPB3 Is Expressed in Various Tissues, and Its Expression Changes in Response to Stress

**Figure 1:**
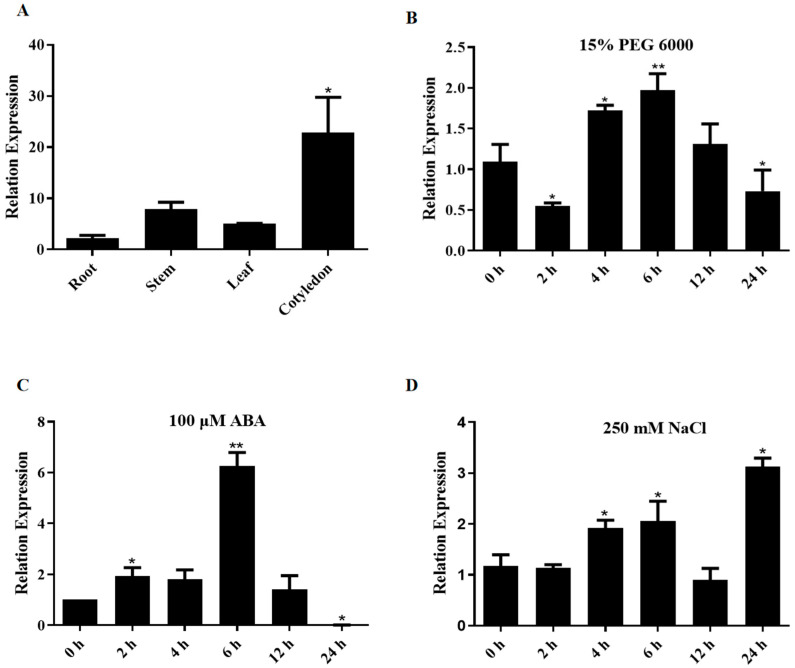
Analysis of *GhNRPB3* expression patterns. (**A**) Changes in gene expression in different tissues, with root tissues used as the control. (**B**) Changes in gene expression from 0–24 h under 15% PEG 6000 treatment conditions. (**C**) Changes in gene expression from 0-24 h under 100 μM ABA treatment conditions. (**D**) Changes in gene expression from 0–24 h under 250 mM NaCl treatment conditions. The error bars represent the means ± SDs. Statistically significant differences compared with the control are denoted by * *p* < 0.05 and ** *p* < 0.01.

The tissue-specific expression of *GhNRPB3* was analyzed by real-time fluorescence quantitative PCR (qPCR). The relative expression level of *GhNRPB3* was greatest in cotyledons (22.8-fold induction), followed by stems (7.9-fold induction) and leaves (5-fold induction), and the expression level of *GhNRPB3* was lowest in root tissues (Figure 1A).

The expression patterns of *GhNRPB3* under various stress conditions were subsequently analyzed by qPCR. When the gene was induced with 15% PEG 6000, its expression was upregulated at 4 h (1.7-fold) and 6 h (1.9-fold) compared with that at 0 h but significantly downregulated at 2 h (0.6-fold) and 24 h (0.7-fold) (Figure 1B). Moreover, 100 μM ABA treatment resulted in a unique response pattern; compared with that at 0 h, expression was significantly induced at 2 h (1.9-fold) and 6 h (6.3-fold), whereas expression was significantly inhibited at 24 h (Figure 1C). Under 250 mM NaCl stress conditions, compared with that at 0 h, the expression level of *GhNRPB3* significantly increased at 4 h (1.9-fold) and 6 h (2.2-fold) and peaked at 24 h (3.2-fold) (Figure 1D).

### 2.2. Silencing of GhNRPB3 Enhances Cotton Drought Resistance

**Figure 2:**
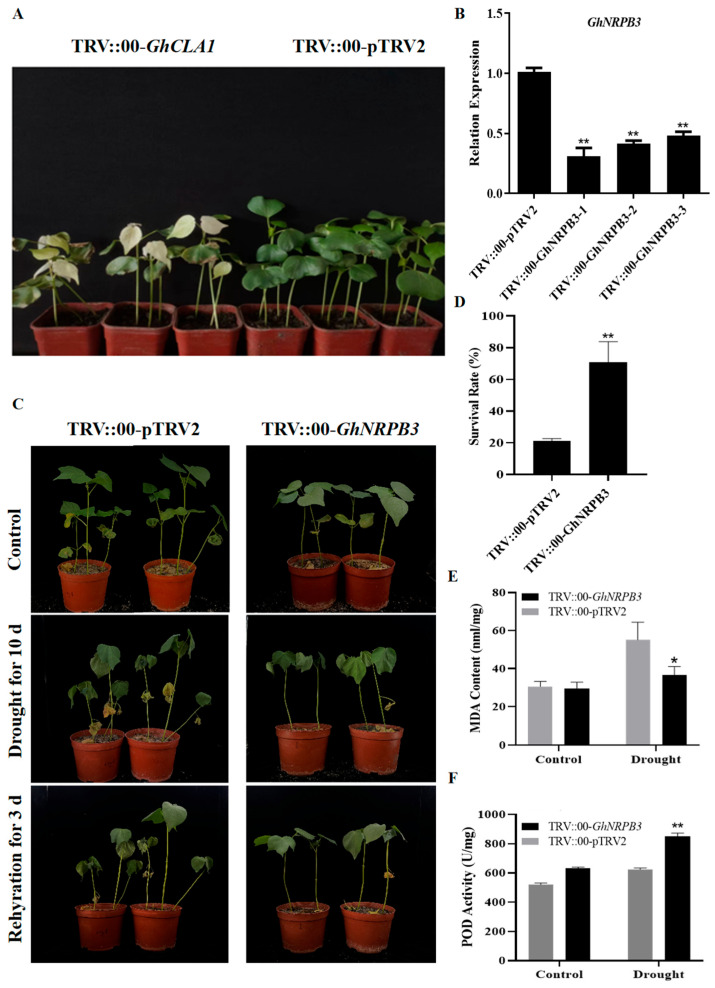
Analysis of drought resistance in cotton with different *GhNRPB3* expression levels. (**A**) White phenotype. (**B**) Assessment of the silencing efficiency of TRV::00-*GhNRPB3*. (**C**) Natural drought treatment. (**D**) Survival rate statistics. (**E**) Malondialdehyde (MDA) content. (**F**) Peroxidase (POD) activity. The error bars represent the means ± SDs. Statistically significant differences compared with the control are denoted by * *p* < 0.05 and ** *p* < 0.01.

After approximately 14 d of cultivation following infection, the TRV::*GhCLA1* cotton plants (positive control group) exhibited a typical chlorophyll-deficient albino phenotype in their true leaf tissues (Figure 2A). qPCR was used to evaluate the efficiency of *GhNRPB3* silencing, and the results revealed that the expression level of the *GhNRPB3* gene in the silenced plants was significantly lower than that in the negative control plants (Figure 2B); these results confirmed that the target gene was successfully silenced by the VIGS system.

To elucidate the function of the *GhNRPB3* gene in the physiology of drought resistance in cotton, TRV::00-*GhNRPB3*-treated and negative control TRV::00-pTRV2-treated plants were subjected to natural drought conditions without a water supply. The results revealed that before drought treatment, there was no difference in phenotype between the TRV::00-*GhNRPB3* and TRV::00-pTRV2 groups (Figure 2C). However, after 10 d of drought stress, the leaves of the TRV::00-pTRV2 plants were more severely wilted, whereas those of the plants in the TRV::00-*GhNRPB3* group grew well (Figure 2C). After the plants were watered again for 3 d, the survival rate of the TRV::00-pTRV2 group was 21.11%, and that of the TRV::00-*GhNRPB3* group was 70.77% (Figure 2D). The malondialdehyde (MDA) content and peroxidase (POD) activity did not significantly differ between the TRV::00-pTRV2 and TRV::00-*GhNRPB3* groups before drought stress treatment. However, after 10 d of stress treatment, the MDA content of the TRV::00-*GhNRPB3* group was significantly lower than that of the TRV::00-pTRV2 group (Figure 2E), and the POD activity was significantly greater than that of the TRV::00-pTRV2 group (Figure 2F). These results revealed that silencing the *GhNRPB3* gene significantly increased the drought resistance of cotton.

### 2.3. Analysis of Drought Stress-Related Gene Expression

**Figure 3:**
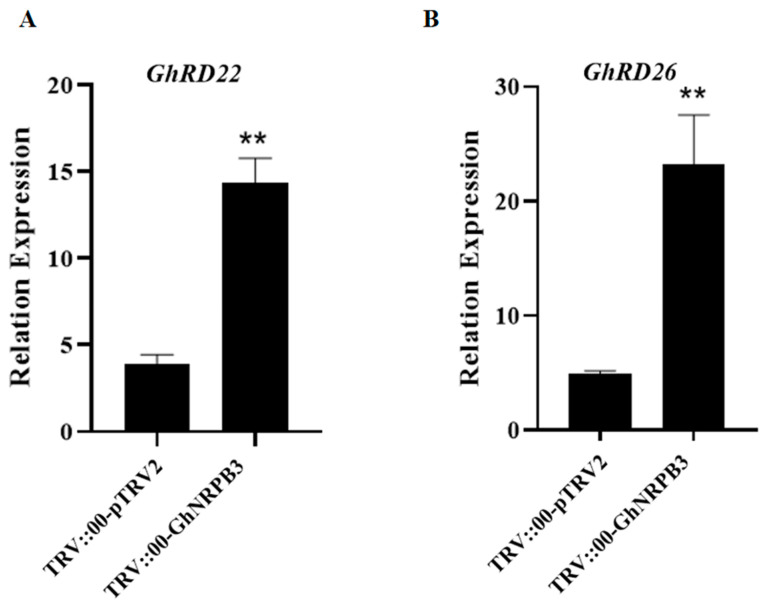
The expression levels of drought stress-responsive genes in *GhNRPB3-*silenced plants under drought stress conditions. (**A**) *GhRD22*. (**B**) *GhRD26*. The error bars represent the means ± SDs. Statistically significant differences compared with the control are denoted by ** *p* < 0.01.

To investigate the regulatory role of *GhNRPB3* in the response to drought stress, qPCR was used to measure the expression levels of the *GhRD22* and *GhRD26* genes in the true leaves of plants from the TRV::00-*GhNRPB3* and TRV::00-pTRV2 groups after 10 d of drought. Compared with those in the TRV::00-pTRV2 group, the expression levels of the drought response marker genes *GhRD22* and *GhRD26* in the leaves of the plants in the TRV::00-*GhNRPB3* group tended to significantly increase (Figure 3).

### 2.4. Silencing of GhNRPB3 Expression Enhances the Salt Tolerance of Cotton

**Figure 4:**
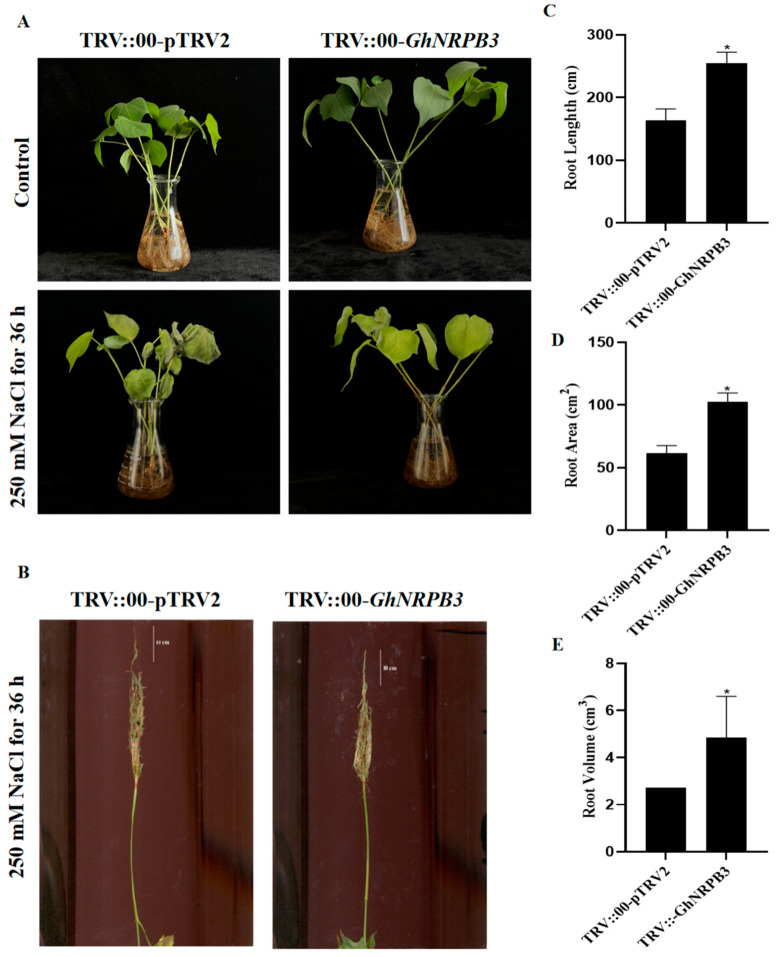
Analysis of the tolerance to salt of cotton plants with different levels of *GhNRPB3* expression. (**A**) Leaf phenotype after treatment with 250 mM NaCl. (**B**) Root phenotype after treatment with 250 mM NaCl. (**C**) Statistical analysis of root length statistics after 36 h of treatment with 250 mM NaCl. (**D**) Statistical analysis of the root area after 36 h of treatment with 250 mM NaCl. (**E**) Statistical analysis of root volume after 36 h of treatment with 250 mM NaCl. The error bars represent the means ± SDs. Statistically significant differences compared with the control are denoted by * *p* < 0.05.

TRV::00-*GhNRPB3*-treated plants (experimental group) and TRV::00-pTRV2-treated plants (control group) were treated with 250 mM NaCl. The results revealed that there was no difference in phenotype between the TRV::00-*GhNRPB3* and TRV::00-pTRV2 groups before the induction of salt stress, and the leaves were elongated (Figure 4A). After being exposed to 250 mM NaCl for 36 h, the plants in the TRV::00-*GhNRPB3* group grew well, whereas the plants in the TRV::00-pTRV2 group exhibited more severe wilting and leaf dehydration (Figure 4A). The results of the determination of root length, root area, and root volume revealed that compared with the plants in the TRV::00-pTRV2 control group, those in the TRV::00-*GhNRPB3* group had longer roots (Figure 4B,C), greater root areas (Figure 4D), and greater root volumes (Figure 4E). These results indicate that the TRV::00-*GhNRPB3*-treated plants exhibited a certain degree of tolerance to salt stress conditions through alterations in their root morphology.

## 3. Discussion

The precise regulation of gene expression is the core mechanism underlying plant growth and development, and the dynamic and flexible regulation of gene expression determines the functional differentiation of cells in specific tissues and developmental stages. Pol II is the core executor of transcriptional regulation, and mutant plants that are deficient in its subunits exhibit a lethal phenotype [16]. Moreover, the T-DNA insertion mutant *nrpb3-2* of the *NRPB3* gene exhibits homozygous lethality, further confirming the irreplaceable biological function of Pol II in plant growth and development [12]. In *Arabidopsis*, the *NRPB3* gene is constitutively expressed, and its expression is significantly upregulated in tissues that undergo vigorous division [12]. Consistent with these findings, the results of this study revealed that the homologous *GhNRPB3* gene in cotton is expressed in tissues such as roots, stems, leaves, and cotyledons, with its level of expression in cotyledon tissues being particularly significant. This conserved expression pattern across species suggests that the NRPB3 subunit may regulate plant growth and development by maintaining the stability and transcriptional activity of Pol II during plant evolution.

In terms of the mechanism through which the Pol II subunit participates in stress responses, different species exhibit unique regulatory characteristics. Previous studies have shown that the expression of the *RPB2* and *ScRpb4* genes usually remains stable and is not easily influenced by the external environment [17,18]. However, in yeast, *RPB4* expression levels are low under normal conditions, but *RPB4* expression is upregulated under stress conditions such as heat shock. RPB4 and RPB7 subsequently form a stable heterodimer and integrate into Pol II, increasing its ability to promote transcriptional elongation. Notably, the overexpression of *RPB7* can partially compensate for the growth defects of *RPB4*-deficient cells under mild temperature stress conditions (such as 34 °C), but *RPB7* cannot function under more stringent high-temperature conditions. These findings indicate that although *RPB7* expression is adaptable to stress, the full function of *RPB7* still depends on the presence of *RPB4* [19]. The protein similarity between GhNRPB3 and RPB4 and RPB7 is only 8.7% and 5.96%, respectively, but it exhibits similar environmental response characteristics to those of RPB4 and RPB7 and its expression is affected by drought, ABA, and NaCl. These findings indicate that some subunits of Pol II may have unique stress response functions in different organisms or under different stress conditions. We found that under 15% PEG 6000-simulated drought stress, its expression was downregulated at 2 h and 24 h, which may reflect an energy-saving mechanism during initial dehydration and the initiation of programmed death under long-term stress, respectively. Its upregulation at 4 h and 6 h reflects the activation of the stress adaptation pathway. Under ABA treatment, rapid induction at 2 h suggests an immediate response to the ABA signaling pathway, with peak expression at 6 h indicating enhanced positive feedback regulation, whereas significant inhibition at 24 h may be due to negative feedback regulation or a change in survival strategy. NaCl stress presents a three-stage response: the initial upregulation (1.9–2.2-fold) at 4–6 h may be related to the activation of the SOS pathway, the downregulation at 12 h reflects metabolic redistribution, and the peak at 24 h (3.2-fold) suggests the initiation of ABA-dependent long-term adaptation mechanisms.

Previous studies have shown that the Pol II subunit NRPB2 participates in regulating stomatal development in plants and that its weak *nrpb2-3* mutation can lead to the generation of a stomatal phenotype in *Arabidopsis* [12]. Similarly, *NRPB3* participates in the regulation of stomatal development. Although *NRPB3* overexpression does not cause significant phenotypic changes, some of its functionally deficient mutants exhibit significantly increased stomatal density and clustered stomatal number [12]. Because stomata are key structures through which plants respond to drought, their density and distribution directly affect their transpiration and water retention capacity. Therefore, the Pol II subunit may indirectly affect plant drought resistance by regulating stomatal development. However, this study revealed that silencing the *GhNRPB3* gene in cotton via VIGS technology can significantly increase plant tolerance to drought and salt. These findings suggest that Pol II subunits may participate in the regulation of adaptation to environmental conditions through different molecular mechanisms in different plants.

The *AtRD22* and *AtRD26* genes of *Arabidopsis* are typical marker genes of the response to abiotic stress and are involved in various stress response processes [20,21]. The results of this study revealed that drought stress induced significant upregulation of the expression of the homologous genes *GhRD22* and *GhRD26* in *GhNRPB3*-silenced cotton plants, indicating that GhNRPB3 may act as a negative regulatory factor to inhibit the activation of these stress-responsive genes.

## 4. Materials and Methods

### 4.1. Plant Materials

In this study, the main upland cotton variety Y1169 in Xinjiang was used as the experimental material. After the surfaces of the seeds were disinfected with a 30% hydrogen peroxide (H_2_O_2_) solution for 4 h, they were rinsed thoroughly with distilled water, placed in a constant-temperature incubator, and cultivated at 28 °C for 2 d to promote germination until they became white. The exposed white seeds were sown in soil containing vermiculite (Shijiazhuang Ruichang Agricultural Technology Co., Ltd., Shijiazhuang, China) and nutrient soil (Pindstrup Mosebrug A/S Co., Ltd., Ryomgård, Denmark). After one week of cultivation under a film cover, the film was removed, and the seeds were cultivated in an artificial climate chamber at Xinjiang Agricultural University (28 °C, 16 h of light/8 h of darkness); when the cotton had grown to the three-leaf stage, the roots, stems, leaves, and cotyledons were collected.

Cotton seedlings that had grown to the three-leaf stage were transferred from the soil pots to a solution containing Hoagland nutrients and allowed to recover for 2 d, after which they were subjected to stress treatment with Hoagland nutrient solution supplemented with 15% PEG-6000, 250 mM NaCl, or 100 µM ABA [22]. Leaf samples were collected at 0 h, 2 h, 4 h, 6 h, 12 h, and 24 h.

### 4.2. Extraction of Total RNA from Cotton and Synthesis of First-Strand cDNA

Total RNA was extracted from cotton using a polysaccharide polyphenol plant total RNA extraction kit (Tiangen Biochemical Technology (Beijing) Co., Ltd., Beijing, China). A TransScript*^®^* First Strand cDNA Synthesis SuperMix Reverse Transcription Kit (Beijing Quanshijin Biotechnology Co., Ltd., Beijing, China) was subsequently used to synthesize first-strand cDNA.

### 4.3. qPCR

A fluorescence quantitative PCR kit from Beijing TransGen Biotech Co., Ltd., Beijing, China, was used to set up 20 μL reactions according to the instructions, and amplification was performed on an ABI 7500 rapid real-time fluorescence quantitative PCR instrument (Applied Biosystems, Foster City, CA, USA). The amplification procedure was conducted according to the instructions provided in the aforementioned reagent kit manual. Using *GhUBQ7* and *AtUBQ3* as internal reference genes for cotton and *Arabidopsis*, respectively, the cycle threshold (Ct) values of the target genes and internal reference genes in each sample were determined. The experimental data were analyzed using the 2^−∆∆Ct^ method, with three biological replicates for each treatment. The sequences of the primers used are shown in Table S1.

### 4.4. VIGS and Gene Expression Assessment

A fragment of the 3′UTR (274 bp) of the *GhNRPB3* gene was ligated to the viral empty vector TRV::00-pTRV2 via the double enzyme digestion method to construct the recombinant vector TRV::00-*GhNRPB3*. The recombinant plasmid was transformed into *Agrobacterium* GV3101 competent cells via the freeze-thaw method. In accordance with previously described methods for the infection of cotton [23], TRV::00-*GhCLA1* was used as a positive control. qPCR was used to measure the efficiency of gene silencing in cotton plants that were treated with TRV::00-*GhNRPB3*. Three biological replicates were analyzed from each treatment group, with no fewer than 15 cotton plants per replicate. The sequences of the primers used are shown in Table S1.

### 4.5. Stress Treatment

With respect to the natural drought treatment, the silenced TRV::00-*GhNRPB3* and control TRV::00-pTRV2 plants that grew consistently at the 3-leaf stage under a normal water supply were selected, and watering was stopped until a phenotypic difference was detected in the cotton plants.

For the 250 mM NaCl treatment, water was supplied normally until the cotton reached the 3-leaf stage, and the watering was stopped for 5 days. Afterward, a 250 mM NaCl solution was applied to the bottom of the flowerpot, and the solution was removed after the soil was soaked, until the TRV::00-*GhNRPB3*-silenced plants and the TRV::00-pTRV2 control plants exhibited phenotypic differences.

The second true leaves of TRV::00-*GhNRPB3*-treated plants and TRV::00-pTRV2-treated control plants were collected before stress treatment (0 d) and after 10 d of stress. A kit from Nanjing Jiancheng Technology Co., Ltd., Nanjing, China, was used to measure POD activity and MDA content according to the manufacturer’s instructions.

An Epson root scanner was used to obtain root images, and WinRHIZO Pro 2017 analysis software was used to process the images and extract the parameters, including root length, root area, and root volume.

### 4.6. Statistical Analysis

All the data were subjected to independent sample t tests and one-way ANOVA with SPSS 17.0 (IBM, New York, NY, USA), and graphs were generated with GraphPad Prism 9.0 (GraphPad Software, San Diego, CA, USA).

## 5. Conclusions

*GhNRPB3*, the third subunit of Pol II, is widely expressed in multiple cotton tissues and may be involved in basic transcriptional regulation. The expression of this gene is induced by abiotic stress, and silencing of *GhNRPB3* expression significantly increased the tolerance of cotton to drought and salt stress, indicating that it negatively regulates the response to both drought and salt stress. This discovery not only expands our understanding of the functional diversity of Pol II subunits in plants but also reveals new candidate genes for improving crop stress resistance via genetic mechanisms. Future research could further explore the specific molecular mechanisms through which GhNRBP3 negatively regulates the stress response, such as by identifying its interaction network under stress conditions through proteomics or by analyzing how it dynamically regulates the transcription of stress-related genes through the Pol II complex, thereby providing more precise targets for the design of stress-resistant crops.

## Data Availability

The original contributions presented in this study are included in the article/Supplementary Material. Further inquiries can be directed to the corresponding author.

## Supplementary Materials

The following supporting information can be downloaded at https://www.mdpi.com/article/10.3390/plants14162575/s1; Table S1: Primer sequences used in this study.
