# Supplementary material for: GhNRPB3 Negatively Regulates Drought and Salt Tolerance in Cotton
Source: Plants (Basel). 2025 Aug 19;14(16):2575. doi: 10.3390/plants14162575 (PMC12389714; doi:10.3390/plants14162575)
Supplement: Supplementary file 1 [file plants-14-02575-s001.zip › plants-3795401-supplementary.pdf]

Table S1 Primer sequences used in this study

**Name**	**Sequence**
GhNRPB3-qF	5'-CATCGACTCGGTCTCATCCC-3'
GhNRPB3-qR	5'-AAGATGGAACTCGACGGAGC-3'
GhUBQ7-F	5'-GACCTACACCAAGCCCAAGAAG-3'
GhUBQ7-R	5'-TGAGCCCACACTTACCACAATAGT-3'
GhNRPB3-TRV2-F	5'-CCGGAATTCCACATGCGAGGAGGTTGA-3'
GhNRPB3-TRV2-R	5'-CGGGATCCACTTCATCCGGTTAAGAGC-3'
GhRD22-F	5'-AGAGGGTGCTGACGGAACAA-3'
GhRD22-R	5'-TGCTTAGGGTTCCATGCGGA-3'
GhRD26-F	5'-GCGAGCTGATAACTCGGGGA-3'
GhRD26-R	5'-GTCGGGCACCGAAACCCATA-3'
